# An improved adaptive position tracking strategy for automatic shift actuator with uncertain parameters

**DOI:** 10.1038/s41598-024-59952-1

**Published:** 2024-04-24

**Authors:** Chengqiang Yin, Shuai Wang, Jie Gao, Guangfei Xu, Jian Wu

**Affiliations:** 1 School of Machinery and Automation, Weifang University, Weifang, 261061 China; 2 School of Mechanical and Automobile Engineering, Liaocheng University, Liaocheng, 252059 China

**Keywords:** Automatic clutch system, Position tracking, Adaptive control, Finite time control, Parameter update, Energy science and technology, Engineering

## Abstract

Realizing precise and fast position control of the gear is a challenging issue because of its nonlinearity, parameter uncertainty and external disturbance. Therefore, this paper researches the clutch position control considering the influence because of the factor on the system performance. By virtue of the traditional adaptive control method, an improved strategy based on finite time theory is proposed to further improve the convergence rate as well as the position tracking precision. First, a model of electromechanical clutch actuator system is established by theoretical analysis. Then, an enhanced adaptive controller is designed using finite time idea by introducing power function in the virtual control. And parameter update rate is adopted in the control action. Next, the stability of the control system is proved theoretically. Finally, Matlab simulations and experimental bench test are carried out to exhibit the effectiveness of the presented method. The results show that the satisfactory performance has been achieved with accurate position tracking and fast convergence speed.

## Introduction

The applications of electromechanical systems in vehicles are more and more extensive with the development of control theory as well as the electronic technology. The automated manual transmission (AMT) is obtained to realize the automatic control of shift by introducing automatic control system to the actuator while keeping the original gear mechanical transmission structure.

In the AMT system, clutch is a critical component. As we know, shifting control strategy and if actuator can accurately execute the control instruction will have effect on the shifting quality, such as shift smoothness, riding comfort and even the clutch life^1^. Because in the clutch engagement process, little wear between the clutch friction plates, smooth position engagement and short shift time are expected. However, the serious nonlinear dynamic characteristics of the transmission and clutch system itself undoubtedly increase difficulties for the clutch control.

According to different design principles, different clutch architectures have been devised such as friction clutch^2,3^, Electro mechanically clutch taking motor as power source^4,5^, hydraulic or electro hydraulic clutch^6,7^ and electro pneumatic clutch^8,9^. To realize clutch engagement or disengagement control, clutch chamber pressure, transmission torque and clutch piston displacement are the mostly used control modes. But no matter what kind of drive actuator is adopted, control with enhanced adaptability and robustness is required to achieve the accurate tracking of the clutch engagement speed or engagement position. Therefor, some researches have been focused on the automatic clutch control. Despite numerous achievements have been obtained on the AMT, optimizations for some problems in the clutch control still are feasible^10^.

No matter what kind of control is used, the accurate position tracking is the basic requirement for the clutch control. The proportional integral derivative (PID) control is widely adopted in engineering because of its simple structure and convenience. But to the process of clutch engagement accompanied with disturbance and uncertainty, it is difficult to achieve rapid and accurate tracking performance for the conventional method. In^11^, feed-forward structure and PID controller were designed to control the speed of the engine and an optimized control method based on LQR was presented for the actuator position. In^12^, the clutch working process based on single neuron adaptive PID was designed, and the non-contact locking cylinder control method for clutch actuator piston was proposed, which could effectively reduce the torque impact and friction work during clutch engagement. Zhou^13^ proposed a control scheme aiming to eliminate position error, in which a logic-switched controller and a single neuron PID controller were integrated to realize position tracking. To control the shift actuator position accurately, a synchronization speed control scheme was proposed based on the sliding mode theory^14^. To realize the servo control of the position tracking of automatic clutch, current-position double closed-loop control system for clutch position tracking was put forward. Udwadia-Kalaba equation was used to solve constraint force without introducing Lagrange multiplier and other parameters^15^. Dong et al.^16^ designed a ball-ramp electromechanical clutch actuator for electric vehicles and established the actuator model. The axial position of the clutch was regarded as the control target to control the torque of the clutch, and triple-step control was adopted to track the clutch position. In^17^, the position controller and the force controller were employed in different stages. Bao et al.^18^ divided the gear shift into three stages and adopted the iterative learning control method to deal with the uncertainties and disturbance in the first stage, and linear quadratic regulator and Takagi and Sugeno (T-S) fuzzy were used in the synchronization stage and post-synchronization stage respectively.

To deal with the disturbances and uncertainties in the clutch system, designing observer or estimator is the mostly adopted manner in the shift control. Such as an output feedback based high gain order observer was designed and a robust recursive controller based on backstepping method was proposed for an electromechanical dry clutch^19^. To track the desired position, Jinsung et al.^20^ devised an adaptive controller and observer to compensate the disk friction and unstructured disturbance, by which the engagement torque could be controlled properly. In^21^, a detailed clutch model was established and a control scheme according to model predictive control and the estimated resistance torque was proposed to control the position of the clutch. For a wet clutch, a pressure tracked control scheme adopting sliding mode control and feed forward was presented in^22^. According to both the estimation of the model uncertainties of the pressure and the state observer, the pressure tracking for the clutch control can be realized. Usually, actuator position can be translated into clutch torque according to the relationship in a position–torque map. In^23^, a clutch observer considering the torque uncertainty was designed according to the friction model to achieve the position control. What’s more, robust control strategies have been adopted to solve the uncertainties in the process of clutch engagement. Bécsi^24^ designed a parameter varying system for an electromechanical clutch actuator, for which model predictive controller and LPV-$H_{\infty }$ position controller were designed. Ouyang et al.^25^ presented a robust control strategy to realize position tracking, in their control scheme, a feedforward controller designed according to transfer function was used to improve the tracking performance, and feedback controller based on $\mu$ synthesis was designed to enhance robustness and disturbance rejection performance.

Shifting time is one of the important indices that determine the torque interruption during the gear-shifting process. Long shifting time will deteriorate the quality of the shift gear. In^26^, to minimize the shifting time, a position control system was devised on the basis of model assisted reduced order for the linear electromagnetic gear-shifting actuator. In^27^, a feedback control strategy was proposed for an AMT system, in which the output of the controller could be accelerated by selecting maximum value or zero in order to control the shifting time. What’s more, Jiang et al.^28^ presented a sliding mode active disturbance rejection control algorithm to resolve the problems of the long shift time and uncertainty. For most of the ordinary control strategy, to obtain fast and steady response in the process of clutch position tracking system is difficult. Finite time control theory has many advantages, such as shortening dynamic response time, rapid convergence speed and strong disturbance rejection ability in comparation with conventional control methods^29,30^.

Motivated by the above achievements, taking merit of the finite time control and adaptive control theory, finite time based adaptive control for clutch position tracking system is developed, which can achieve fast convergence as well as accurate position tracking. Compared with other methods, the advantages of the control scheme for the clutch actuator proposed are as follows

1. A control model combining the brush motor with the ball screw in the actuator is established. On the basis of the model, the control of the shifting fork and the shifting operation can be realized steady by adjusting the displacement of the ball screw.
2. Parameter update rate is devised in the adaptive control theory to weaken the impact of parameter changes on shifting operation, by which unknown disturbance of parameters in the shifting system is resolved.
3. By virtue of the finite time control method, the finite time adaptive control gives a closed loop system with faster response and higher tracking precision for the shifting operation. Therefore, excellent quality of shift gear can be achieved with less abruption.

For clear illustration, organization of this paper is arranged as: Models for clutch actuator system are established in Section "System modeling". Clutch position tracking control strategy is detailed in Section "Finite time adaptive controller design". And system stability is analyzed in Section "Stability analysis". The validity of the presented control method is illustrated in Section "Simulation and test results" and conclusions are given in the last section.

## System modeling

**Figure 1:**
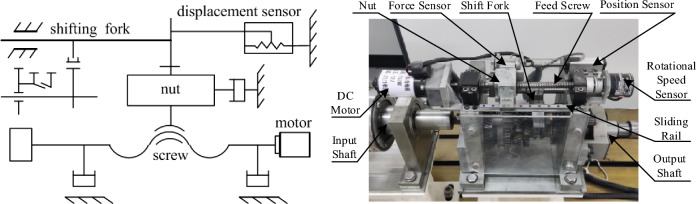
Shifting actuator diagram.

The research object of this paper is the two-gear AMT in the laboratory, the shift actuator is shown in Fig. 1, which is mainly composed of shift motor, ball screw mechanism, shift fork and line displacement sensor. Motor is adopted as the power source, and power conversion unit is selected as ball screw, in which the ball is connected with the screw and nut to transfer the power. The rotational motion of the motor is transformed into linear motion by the ball screw, and the fork is pushed to realize gear shift. The linear displacement sensor is adopted to obtain the displacement of the fork during shift process.

For the clutch actuator, shift motor and ball screw are the main control objects, and the models for them will be established. Because the torque from the shifting motor is regarded as the torque input to the ball screw^31^. Therefore, a new integrated model is desired to be built according to the torque by establishing the torque balance equation for both the shift motor and the ball screw.

### Shift motor modeling

**Figure 2:**
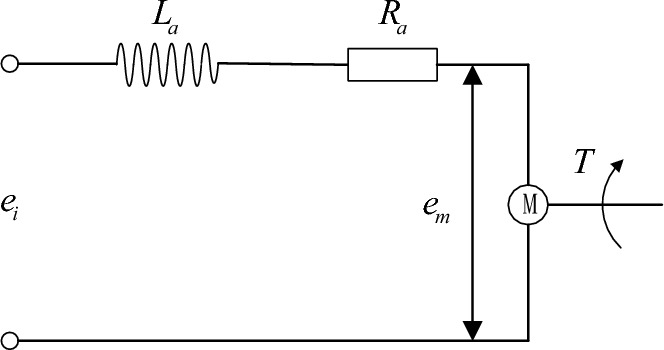
Schematic diagram of brushed DC motor.

The permanent magnet brushless DC motor is adopted to in the shift actuator for the electric automatic clutch. Compared with the AC motor, the DC motor is characterized as small size, high efficiency and long life. It is widely used in the shifting control for the clutch. The schematic diagram of the DC motor is given in Fig. 2.

The voltage balance equation and torque balance equation for the permanent magnet brushed DC motor can be obtained according to the Kirchhoff’s voltage law and Newton’s third law.

1 $$\left\{ {\begin{array}{*{20}l} {uV_{bat} = L_{m} \dot{i}_{m} + R_{m} i_{m} + K_{e} \omega_{m} } \hfill \\ {K_{T} i_{m} + T_{L} = I_{m} \dot{\omega }_{m} } \hfill \\ \end{array} } \right.$$

### Ball screw modeling

According to the structure of the adopted clutch actuator, the input torque of the ball screw is equal to the output torque of the brushed DC motor, part of which is used to overcome the resistance moment generated by the moment of inertia of the wire, the translational inertia of the nut and the inertia moment of the ball movement, and the other part is used to overcome the friction resistance torque and equivalent resistance moment on the nut produced by the shift fork. Then the torque balance equation of the ball screw is established as

2 $$T_{L} = J_{s} \frac{d\omega }{{dt}} + m\frac{dv}{{dt}} + T_{f}$$

where, *T*_*L*_ denoted the output torque of the DC motor, $J_{s}$ denotes the moment of inertia of ball screw and coupling bolt, $\omega$ denotes the angular velocity of the ball screw, $m$ denotes the mass of the nut, $v$ denotes the translational speed of the nut, $T_{f}$ denotes the friction moment of the nut.

The equation for the thrust produced by the ball screw is

3 $$F = \frac{{2T_{f} }}{{d\tan \left( {\beta + \varphi } \right)}}$$

where, $F$ is the resistance acting on the nut by the shift fork, $d$ is the pitch diameter of the thread, $\beta$ is the friction angle, $\varphi$ is the lead angle.

The function relation between the angular velocity of the ball screw and the translational movement of the nut during the working process is as follow

4 $$\frac{2\pi }{\omega } = \frac{L}{v}$$

where, $L$ denotes the lead of the ball screw, $\omega$ denotes the angular velocity of the motor, $v$ denotes the translational velocity of the ball screw. Juggling the above four equations, we can obtain

5 $$T_{L} = \left( {J_{s} + \frac{mL}{{2\pi }}} \right)\frac{d\omega }{{dt}} + \frac{1}{2}Fd\tan \left( {\beta + \varphi } \right)$$

### System state space

Substituting Eq. (5) into the brush motor model, we can get as

6 $$\left\{ {\begin{array}{*{20}l} {uV_{bat} = R_{a} i_{a} + L_{a} \dot{i}_{a} + K_{e} \omega_{m} } \hfill \\ {K_{T} i_{a} = J_{equ} \dot{\omega }_{m} + B_{equ} \omega_{m} + \left( {J_{s} + \frac{mL}{{2\pi }}} \right)\frac{d\omega }{{dt}} + \frac{1}{2}Fd\tan \left( {\beta + \varphi } \right)} \hfill \\ \end{array} } \right.$$

At the same time, we can get the following equations from Eq. (4)

7 $$\left\{ {\begin{array}{*{20}c} {v_{s} = \frac{h}{2\pi }\omega_{m} } \\ {a_{s} = \frac{h}{2\pi }\dot{\omega }_{m} } \\ \end{array} } \right.$$

8 $$\begin{gathered} \dot{a}_{s} = \frac{h}{2\pi }\ddot{\omega }_{m} = \frac{h}{2\pi }\left\{ \begin{gathered} \frac{{K_{T} }}{{J_{equ} + J_{s} + \frac{ml}{{2\pi }}}}\left[ \begin{gathered} \frac{{V_{bat} }}{{L_{a} }}u - \frac{{R_{a} }}{{L_{a} }}\left( \begin{gathered} \frac{{J_{equ} + J_{s} + \frac{ml}{{2\pi }}}}{{K_{T} }}\left( {\frac{2\pi }{h}a_{s} } \right) + \hfill \\ \frac{{B_{equ} }}{{K_{T} }}\left( {\frac{2\pi }{h}v_{s} } \right) + \frac{{Fd\tan \left( {\varphi + \beta } \right)}}{{2K_{T} }} \hfill \\ \end{gathered} \right) \hfill \\ - \frac{{K_{e} }}{{L_{a} }}\left( {\frac{2\pi }{h}v_{s} } \right) \hfill \\ \end{gathered} \right] \hfill \\ - \frac{{B_{equ} }}{{J_{equ} + J_{s} + \frac{ml}{{2\pi }}}}\left( {\frac{2\pi }{h}v_{s} } \right) + \frac{{\dot{F}d\tan \left( {\varphi + \beta } \right)}}{{2\left( {J_{equ} + J_{s} + \frac{ml}{{2\pi }}} \right)}} \hfill \\ \end{gathered} \right\} \\ = \frac{{hK_{T} V_{bat} }}{{2\pi L_{a} \left( {J_{equ} + J_{s} + \frac{ml}{{2\pi }}} \right)}}u - \frac{{R_{a} }}{{L_{a} }}a_{s} - \frac{1}{{J_{equ} + J_{s} + \frac{ml}{{2\pi }}}}\left( { - \frac{{R_{a} B_{equ} }}{{L_{a} }} - \frac{{K_{T} K_{e} }}{{L_{a} }} - B_{equ} } \right)v_{s} - \frac{{hR_{a} Fd\tan \left( {\varphi + \beta } \right)}}{{4\pi L_{a} \left( {J_{equ} + J_{s} + \frac{ml}{{2\pi }}} \right)}} + \frac{{\dot{F}dh\tan \left( {\varphi + \beta } \right)}}{{4\pi \left( {J_{equ} + J_{s} + \frac{ml}{{2\pi }}} \right)}} \\ \end{gathered}$$

The shifting force is complex while the shifting action is executed, what’s more, the clearance and friction between the parts in the shift clutch can’t be measured accurately. Therefore, the shifting force is characterized as obvious uncertainty and nonlinear, and it is usually regarded as unknown disturbance in the control^32^.

Selecting the displacement, velocity and acceleration of ball screw as the state variables for the control system, that is $x_{1} = x_{{\text{s}}} ,x_{2} = v_{{\text{s}}} ,x_{3} = a_{{\text{s}}}$ and selecting the displacement of ball screw as the output of the system, the state space description for the clutch actuator can be established as

9 $$\left\{ {\begin{array}{*{20}l} {\dot{x}_{1} = x_{2} } \hfill \\ {\dot{x}_{2} = x_{3} } \hfill \\ {\dot{x}_{3} = a_{1} x_{2} + a_{2} x_{3} + bu + d} \hfill \\ {y = x_{1} } \hfill \\ \end{array} } \right.$$

where

10 $$\begin{gathered} a_{1} = - \frac{1}{{J_{equ} + J_{s} + \frac{ml}{{2\pi }}}}\left( { - \frac{{R_{a} B_{equ} }}{{L_{a} }} - \frac{{K_{T} K_{e} }}{{L_{a} }} - B_{equ} } \right) \hfill \\ a_{2} = - \frac{{R_{a} }}{{L_{a} }},b = \frac{{hK_{T} V_{bat} }}{{2\pi L_{a} \left( {J_{equ} + J_{s} + \frac{ml}{{2\pi }}} \right)}} \hfill \\ d = - \frac{{hR_{a} Fd\tan \left( {\varphi + \beta } \right)}}{{4\pi L_{a} \left( {J_{equ} + J_{s} + \frac{ml}{{2\pi }}} \right)}} + \frac{{\dot{F}dh\tan \left( {\varphi + \beta } \right)}}{{4\pi \left( {J_{equ} + J_{s} + \frac{ml}{{2\pi }}} \right)}} \hfill \\ \end{gathered}$$

## Finite time adaptive controller design

### Lemma 1^33^

Consider the system $\dot{x} = f\left( x \right)$, $f\left( 0 \right) = 0$. If there exists continuously differentiable function $V$ which satisfies the following

(1) $V$ is a positive definite function.
There exist $\alpha > 0,\,\beta > 0$, $\gamma \in \left( {0,1} \right)$ and an open neighborhood containing the origin $U \in U_{0}$ such that $\dot{V} \le - \alpha V - \beta V^{\gamma } + c$, then the system is fast finite time stable, and the settling time satisfies $T \le \frac{1}{{\alpha \left( {1 - \gamma } \right)}}\ln \frac{{\alpha V_{0}^{1 - \gamma } + \beta }}{\beta }$
  And the system is globally fast finite time stable if $U = U_{0} = R^{n}$.

As can be seen from Eq. (9) that the established model of the shift gear actuator is a third-order system. So based on the backstepping control theory, the design for the finite time adaptive controller can be divided into three steps.

The error variables used in the control are defined as

11 $$\left\{ {\begin{array}{*{20}c} {z_{1} = x_{1} - x_{d} } \\ {z_{2} = x_{2} - \alpha_{1} } \\ {z_{3} = x_{3} - \alpha_{2} } \\ \end{array} } \right.$$

In the above equations, *x*_*d*_ denotes the desired position of ball screw, $\alpha_{1}$, $\alpha_{2}$ are the virtual desired control signal.

Step 1: On the basis of Eq. (11), we can obtain the following equation

12 $$\dot{z}_{1} = \dot{x}_{1} - \dot{x}_{d} = z_{2} - \alpha_{1} - \dot{x}_{d}$$

Virtual error variable $\alpha_{1}$ is defined as

13 $$\alpha_{1} = - k_{1} z_{1}^{2\beta - 1} - s_{1} z_{1} + \dot{x}_{d}$$

where $k_{1}$, $s_{1}$ and $\beta$ are the positive parameters to be determined. The power function about the error $z_{1}$ introduced into the virtual control rate can make the response converge in finite time by adjusting the parameters $k_{1}$ and $\beta$ in comparison with the conventional adaptive control method.

The Lyapunov function for the first system is defined as

14 $$V_{1} = \frac{1}{2}z_{1}^{2}$$

The derivative of $V_{1}$ can be obtained according to the Eqs. (12) and (13)

15 $$\begin{gathered} V_{1} = z_{1} \dot{z}_{1} \\ = z_{1} \left( {z_{2} + \alpha_{1} - \dot{x}_{d} } \right) \\ = - k_{1} z_{1}^{2\beta } - s_{1} z_{1}^{2} + z_{1} z_{2} \\ \end{gathered}$$

Step 2: The third error variable is introduced into the second equation to solve the control law $z_{3} = x_{3} - \alpha_{2}$.

Further, the following equation can be obtained according to Eqs. (11) and (13).

16 $$\begin{gathered} \dot{z}_{2} = \dot{x}_{2} - \dot{\alpha }_{1} \\ = x_{3} - \left( { - k_{1} \left( {2\beta - 1} \right)z_{1}^{2\beta - 2} \dot{z}_{1} - s_{1} \dot{z}_{1} + \ddot{x}_{d} } \right) \\ = z_{3} + \alpha_{2} + \left( {k_{1} \left( {2\beta - 1} \right)z_{1}^{2\beta - 2} - s_{1} } \right)\left( {x_{2} - \dot{x}_{d} } \right) - \ddot{x}_{d} \\ = z_{3} + \alpha_{2} + \left( {k_{1} \left( {2\beta - 1} \right)z_{1}^{2\beta - 2} - s_{1} } \right)x_{2} \\ - \left( {k_{1} \left( {2\beta - 1} \right)z_{1}^{2\beta - 2} - s_{1} } \right)\dot{x}_{d} - \ddot{x}_{d} \\ \end{gathered}$$

Virtual error variable $\alpha_{2}$ is defined as

17 $$\begin{gathered} \alpha_{2} = - k_{2} z_{2}^{2\beta - 1} - s_{2} z_{2} - z_{1} \\ - \left( {k_{1} \left( {2\beta - 1} \right)z_{1}^{2\beta - 2} - s_{1} } \right)x_{2} \\ + \left( {k_{1} \left( {2\beta - 1} \right)z_{1}^{2\beta - 2} - s_{1} } \right)\dot{x}_{d} + \ddot{x}_{d} \\ \end{gathered}$$

where, $k_{2}$, $s_{2}$ are the positive parameter to be determined.

The Lyapunov function for the second system is defined as

18 $$V_{2} = V_{1} + \frac{1}{2}z_{2}^{2}$$

The derivative of $V_{2}$ can be obtained according to the Eqs. (15) and (16)

19 $$\begin{gathered} \dot{V}_{2} = \dot{V}_{1} + z_{2} \dot{z}_{2} \\ = - k_{1} z_{1}^{2\beta } - s_{1} z_{1}^{2} + z_{1} z_{2} + z_{2} \left[ \begin{gathered} z_{3} + \alpha_{2} + \left( {k_{1} \left( {2\beta - 1} \right)z_{1}^{2\beta - 2} - s_{1} } \right)x_{2} \hfill \\ - \left( {k_{1} \left( {2\beta - 1} \right)z_{1}^{2\beta - 2} - s_{1} } \right)\dot{x}_{d} - \ddot{x}_{d} \hfill \\ \end{gathered} \right] \\ \end{gathered}$$

Step 3:We can conclude from Eqs. (12), (13) and (17) that $\alpha_{2}$ is a function about $x_{1} ,x_{2} ,x_{d} ,\dot{x}_{d} ,\ddot{x}_{d}$, so the following equation can be obtained as

20 $$\begin{gathered} \dot{z}_{3} = \dot{x}_{3} - \dot{\alpha }_{2} \\ = a_{1} x_{2} + a_{2} x_{3} + bu + d \\ - \left( {\frac{{\partial \alpha_{2} }}{{\partial x_{1} }}\dot{x}_{1} + \frac{{\partial \alpha_{2} }}{{\partial x_{2} }}\dot{x}_{2} + \frac{{\partial \alpha_{2} }}{{\partial x_{d} }}\dot{x}_{d} + \frac{{\partial \alpha_{2} }}{{\partial \dot{x}_{d} }}\ddot{x}_{d} + \frac{{\partial \alpha_{2} }}{{\partial \ddot{x}_{d} }}\dddot x_{d} } \right) \\ \end{gathered}$$

Let $$\kappa = \frac{1}{b}$$, $$u = \hat{\kappa }\overline{u}$$

21 $$\begin{gathered} \overline{u} = - \hat{a}_{1} x_{2} - \hat{a}_{2} x_{3} - \tanh \left( {\frac{{z_{3} }}{{\varepsilon_{2} }}} \right)\hat{D} - z_{2} \\ + \left( {\frac{{\partial \alpha_{2} }}{{\partial x_{1} }}\dot{x}_{1} + \frac{{\partial \alpha_{2} }}{{\partial x_{2} }}\dot{x}_{2} + \frac{{\partial \alpha_{2} }}{{\partial x_{d} }}\dot{x}_{d} + \frac{{\partial \alpha_{2} }}{{\partial \dot{x}_{d} }}\ddot{x}_{d} + \frac{{\partial \alpha_{2} }}{{\partial \ddot{x}_{d} }}\dddot x_{d} } \right) - k_{3} z_{3}^{2\beta - 1} - s_{3} z_{3} \\ \end{gathered}$$

where, $k_{3}$, $s_{3}$ are the positive parameters to be determined. $\hat{\kappa }$, $\hat{a}_{1}$, $\hat{a}_{2}$ and $\hat{D}$ are the estimations for $\kappa$, $a_{1}$, $a_{2}$, $D$ respectively.

The update rates for the four estimated parameters are designed as^34^

22 $$\dot{\hat{a}}_{1} = \gamma_{1} x_{2} z_{3} + \gamma_{1} k_{4} \tilde{a}_{1}^{2\beta - 1} + \gamma_{1} s_{4} \tilde{a}_{1}$$

23 $$\dot{\hat{a}}_{2} = \gamma_{2} x_{3} z_{3} + \gamma_{2} k_{5} \tilde{a}_{2}^{2\beta - 1} + \gamma_{2} s_{5} \tilde{a}_{2}$$

24 $$\dot{\hat{\kappa }} = - \gamma_{3} z_{3} \overline{u} + \gamma_{3} k_{6} \tilde{\kappa }^{2\beta - 1} + \gamma_{3} s_{6} \tilde{\kappa }$$

25 $$\dot{\hat{D}} = \gamma_{4} z_{3} \tanh \left( {\frac{{z_{3} }}{\varepsilon }} \right) + \gamma_{4} k_{7} \tilde{D}^{2\beta - 1} + \gamma_{4} s_{7} \tilde{D}$$

where, $\gamma_{1} ,\gamma_{2} ,\gamma_{3} ,\gamma_{4}$, $k_{4} ,k_{5} ,k_{6} ,k_{7}$ and $s_{4} ,s_{5} ,s_{6} ,s_{7}$ are the positive parameters for the update rates. Estimation for these parameters by designing the update rates can decrease the influences because of the model uncertainties and parametric variation of models caused by external factors.

The Lyapunov function for this step is defined as

26 $$V_{3} = V_{2} + \frac{1}{2}z_{3}^{2} + \frac{1}{{2\gamma_{1} }}\tilde{a}_{1}^{2} + \frac{1}{{2\gamma_{2} }}\tilde{a}_{2}^{2} + \frac{b}{{2\gamma_{3} }}\tilde{\kappa }^{2} + \frac{1}{{2\gamma_{4} }}\tilde{D}^{2}$$

where,$$\tilde{a}_{1} = a_{1} - \hat{a}_{1} ,\tilde{a}_{2} = a_{2} - \hat{a}_{2} ,\tilde{\kappa } = \kappa - \hat{\kappa },\tilde{D} = D - \hat{D}$$.

The following equation can be obtained from Eqs. (18)–(20)

27 $$\begin{gathered} \dot{V}_{3} = \dot{V}_{2} + z_{3} \dot{z}_{3} - \frac{1}{{\gamma_{1} }}\tilde{a}_{1} \dot{\hat{a}}_{1} - \frac{1}{{\gamma_{2} }}\tilde{a}_{2} \dot{\hat{a}}_{2} - \frac{b}{{\gamma_{3} }}\tilde{\kappa }\dot{\hat{\kappa }} - \frac{1}{{\gamma_{4} }}\tilde{D}\dot{\hat{D}} \\ = - k_{1} z_{1}^{2\beta } - k_{2} z_{2}^{2\beta } - s_{1} z_{1}^{2} - s_{2} z_{2}^{2} + z_{2} z_{3} \\ + z_{3} \left\{ \begin{gathered} a_{1} x_{2} + a_{2} x_{3} + bu + d \hfill \\ - \left( {\frac{{\partial \alpha_{2} }}{{\partial x_{1} }}\dot{x}_{1} + \frac{{\partial \alpha_{2} }}{{\partial x_{2} }}\dot{x}_{2} + \frac{{\partial \alpha_{2} }}{{\partial x_{d} }}\dot{x}_{d} + \frac{{\partial \alpha_{2} }}{{\partial \dot{x}_{d} }}\ddot{x}_{d} + \frac{{\partial \alpha_{2} }}{{\partial \ddot{x}_{d} }}\dddot x_{d} } \right) \hfill \\ \end{gathered} \right\} \\ - \frac{1}{{\gamma_{1} }}\tilde{a}_{1} \dot{\hat{a}}_{1} - \frac{1}{{\gamma_{2} }}\tilde{a}_{2} \dot{\hat{a}}_{2} - \frac{b}{{\gamma_{3} }}\tilde{\kappa }\dot{\hat{\kappa }} - \frac{1}{{\gamma_{4} }}\tilde{D}\dot{\hat{D}} \\ \end{gathered}$$

Substituting Eq. (21) into Eq. (27), we can get

28 $$\begin{gathered} \dot{V}_{3} = - k_{1} z_{1}^{2\beta } - k_{2} z_{2}^{2\beta } - s_{1} z_{1}^{2} - s_{2} z_{2}^{2} + z_{2} z_{3} + a_{1} x_{2} z_{3} + a_{2} x_{3} z_{3} \\ + z_{3} b\kappa \left( \begin{gathered} - \hat{a}_{1} x_{1} - \hat{a}_{2} x_{2} - \tanh \left( {\frac{{z_{3} }}{{\varepsilon_{2} }}} \right)\hat{D} - z_{2} \hfill \\ + \left( {\frac{{\partial \alpha_{2} }}{{\partial x_{1} }}\dot{x}_{1} + \frac{{\partial \alpha_{2} }}{{\partial x_{2} }}\dot{x}_{2} + \frac{{\partial \alpha_{2} }}{{\partial x_{d} }}\dot{x}_{d} + \frac{{\partial \alpha_{2} }}{{\partial \dot{x}_{d} }}\ddot{x}_{d} + \frac{{\partial \alpha_{2} }}{{\partial \ddot{x}_{d} }}\dddot x_{d} } \right) \hfill \\ - k_{3} z_{3}^{2\beta - 1} - s_{3} z_{3} \hfill \\ \end{gathered} \right) \\ \end{gathered}$$

$$- z_{3} b\tilde{\kappa }\overline{u} + z_{3} d - z_{3} \left( \begin{gathered} \frac{{\partial \alpha_{2} }}{{\partial x_{1} }}\dot{x}_{1} + \frac{{\partial \alpha_{2} }}{{\partial x_{2} }}\dot{x}_{2} + \frac{{\partial \alpha_{2} }}{{\partial x_{d} }}\dot{x}_{d} \hfill \\ + \frac{{\partial \alpha_{2} }}{{\partial \dot{x}_{d} }}\ddot{x}_{d} + \frac{{\partial \alpha_{2} }}{{\partial \ddot{x}_{d} }}\dddot x_{d} \hfill \\ \end{gathered} \right) - \frac{1}{{\gamma_{1} }}\tilde{a}_{1} \dot{\hat{a}}_{1} - \frac{1}{{\gamma_{2} }}\tilde{a}_{2} \dot{\hat{a}}_{2} - \frac{b}{{\gamma_{3} }}\tilde{\kappa }\dot{\hat{\kappa }} - \frac{1}{{\gamma_{4} }}\tilde{D}\dot{\hat{D}}$$

$$\begin{gathered} = - k_{1} z_{1}^{2\beta } - k_{2} z_{2}^{2\beta } - k_{3} z_{3}^{2\beta } - s_{1} z_{1}^{2} - s_{2} z_{2}^{2} - s_{3} z_{3}^{2} \\ + a_{1} x_{2} z_{3} + a_{2} x_{3} z_{3} - \hat{a}_{1} x_{2} z_{3} - \hat{a}_{2} x_{3} z_{3} - z_{3} \tanh \left( {\frac{{z_{3} }}{{\varepsilon_{2} }}} \right)\hat{D} - \\ z_{3} b\tilde{\kappa }\overline{u} + z_{3} d - \frac{1}{{\gamma_{1} }}\tilde{a}_{1} \dot{\hat{a}}_{1} - \frac{1}{{\gamma_{2} }}\tilde{a}_{2} \dot{\hat{a}}_{2} - \frac{b}{{\gamma_{3} }}\tilde{\kappa }\dot{\hat{\kappa }} - \frac{1}{{\gamma_{4} }}\tilde{D}\dot{\hat{D}} \\ \end{gathered}$$

## Stability analysis

Equation (28) can be simplified according to Eqs. (22)–(27)

29 $$\begin{gathered} \dot{V}_{3} = \sum\limits_{i = 1}^{3} { - k_{i} z_{i}^{2\beta } } + \sum\limits_{i = 1}^{3} { - s_{i} z_{i}^{2} } + \tilde{a}_{1} x_{2} z_{3} + \tilde{a}_{2} x_{3} z_{3} + z_{3} d - z_{3} \tanh \left( {\frac{{z_{3} }}{\varepsilon }} \right)\left( {D - \tilde{D}} \right) - \\ z_{3} b\tilde{\kappa }\overline{u} + z_{3} d - \frac{1}{{\gamma_{1} }}\tilde{a}_{1} \dot{\hat{a}}_{1} - \frac{1}{{\gamma_{2} }}\tilde{a}_{2} \dot{\hat{a}}_{2} - \frac{b}{{\gamma_{3} }}\tilde{\kappa }\dot{\hat{\kappa }} - \frac{1}{{\gamma_{4} }}\tilde{D}\dot{\hat{D}} \\ \le \sum\limits_{i = 1}^{3} { - k_{i} z_{i}^{2\beta } } + \sum\limits_{i = 1}^{3} { - s_{i} z_{i}^{2} } - z_{3} b\tilde{\kappa }\overline{u} + \tilde{a}_{1} x_{2} z_{3} + \tilde{a}_{2} x_{3} z_{3} + \left[ {\left| {z_{3} } \right| - z_{3} \tanh \left( {\frac{{z_{3} }}{\varepsilon }} \right)} \right]D \\ + z_{3} \tanh \left( {\frac{{z_{3} }}{{\varepsilon_{2} }}} \right)\tilde{D} - \frac{1}{{\gamma_{1} }}\tilde{a}_{1} \dot{\hat{a}}_{1} - \frac{1}{{\gamma_{2} }}\tilde{a}_{2} \dot{\hat{a}}_{2} - \frac{b}{{\gamma_{3} }}\tilde{\kappa }\dot{\hat{\kappa }} - \frac{1}{{\gamma_{4} }}\tilde{D}\dot{\hat{D}} \\ = \sum\limits_{i = 1}^{3} { - k_{i} z_{i}^{2\beta } } + \sum\limits_{i = 1}^{3} { - s_{i} z_{i}^{2} } + \frac{1}{{\gamma_{1} }}\left( { - \dot{\hat{a}}_{1} + \gamma_{1} x_{2} z_{3} } \right)\tilde{a}_{1} + \frac{1}{{\gamma_{2} }}\left( { - \dot{\hat{a}}_{2} + \gamma_{2} x_{3} z_{3} } \right)\tilde{a}_{2} + \frac{b}{{\gamma_{3} }}\left( { - \dot{\hat{\kappa }} - \gamma_{3} z_{3} \overline{u}} \right)\tilde{\kappa } \\ + \frac{1}{{\gamma_{4} }}\left( { - \dot{\hat{D}} + \gamma_{4} z_{3} \tanh \left( {\frac{{z_{3} }}{{\varepsilon_{2} }}} \right)} \right)\tilde{D} + \left[ {\left| {z_{3} } \right| - z_{3} \tanh \left( {\frac{{z_{3} }}{\varepsilon }} \right)} \right]D \\ \end{gathered}$$

According to the document^35^, the following condition can be draw

30 $$0 \le \left| \varsigma \right| - \varsigma \tanh \left( {\frac{\varsigma }{\varepsilon }} \right) \le 0.2785\varepsilon$$

Selecting

31 $$V = V_{3} = \frac{1}{2}z_{1}^{2} + \frac{1}{2}z_{2}^{2} + \frac{1}{2}z_{3}^{2} + \frac{1}{{2\gamma_{1} }}\tilde{a}_{1}^{2} + \frac{1}{{2\gamma_{2} }}\tilde{a}_{2}^{2} + \frac{b}{{2\gamma_{3} }}\tilde{\kappa }^{2} + \frac{1}{{2\gamma_{4} }}\tilde{D}^{2}$$

Substituting Eqs. (22)–(25) into Eq. (29), we can get the following inequation

32 $$\begin{gathered} \dot{V}_{3} \le - k_{1} z_{1}^{2\beta } - k_{2} z_{2}^{2\beta } - k_{3} z_{3}^{2\beta } - k_{4} \tilde{a}_{1}^{2\beta } - k_{5} \tilde{a}_{2}^{2\beta } \\ - bk_{6} \tilde{\kappa }^{2\beta } - k_{7} \tilde{D}^{2\beta } - s_{1} z_{1}^{2} - s_{2} z_{2}^{2} - s_{3} z_{3}^{2} \\ - s_{4} \tilde{a}_{1}^{2} - s_{5} \tilde{a}_{2}^{2} - bs_{6} \tilde{\kappa }^{2} - s_{7} \tilde{D}^{2} + 0.2785\varepsilon \\ \le - mV - nV^{\beta } + \varphi \\ \end{gathered}$$

The relevant parameters can be set according to the method in document^29^

33 $$\begin{gathered} m = \min \left[ {2s_{1} ,2s_{2} ,2s_{3} ,2\gamma_{1} s_{4} ,2\gamma_{2} s_{5} ,2\gamma_{3} s_{6} ,2\gamma_{4} s_{7} } \right] \hfill \\ n = \min \left[ {k_{1} 2^{\beta } ,k_{2} 2^{\beta } ,k_{3} 2^{\beta } ,k_{4} \left( {2\gamma_{1} } \right)^{\beta } ,k_{5} \left( {2\gamma_{2} } \right)^{\beta } ,k_{6} \left( {b^{\beta - 1} } \right)\left( {2\gamma_{3} } \right)^{\beta } ,k_{7} \left( {2\gamma_{4} } \right)^{\beta } } \right] \hfill \\ \varphi = 0.2785\varepsilon \hfill \\ \end{gathered}$$

According to Eqs. (14), (18) and (32), we can see that $V_{i}$ is bounded because $z_{i}$ and $\tilde{a}_{i}$ are all bounded, and the signals in the closed loop system are bounded.

In the meantime, the designed $V$ is positive definite functions and the parameters $s_{j} ,k_{j} ,(j = 1{ - }7)$, $\gamma_{j} (j = 1{ - }4)$ are positive. So according to the Lemma 1, there exist positive parameters $m$, $n$, $\varphi$ and $\beta \in \left( {0,1} \right)$, which makes $\dot{V} \le - mV - nV^{\beta } + \varphi$ a reality, then the system is fast finite time stable. So the tracking error of the position control system can converge to a sufficiently small neighborhood around the origin by selecting proper values of $m,n$.

## Simulation and test results

To demonstrate the effectiveness of the designed finite time adaptive control strategy for the gear shifting actuator, simulations and analyses are provided in this section. What’s more, conventional adaptive control strategy is introduced to illustrate the superior performance of the proposed control scheme. In the simulation, the control parameters of the finite time adaptive controller are the same as those of the adaptive controller, that are $s_{1} = 1$, $s_{2} = 20$, $s_{3} = 12$, $s_{4} = s_{5} = s_{6} = s_{7} = 0.8$, $k_{1} = 13$, $k_{2} = 2000$, $k_{3} = 2000$, $k_{4} = k_{5} = k_{6} = k_{7} = 1000$, $\gamma_{1} = \gamma_{2} = \gamma_{3} = \gamma_{4} = 0.5$, $\varepsilon = 2$, $\beta = 0.9$.

**Figure 3:**
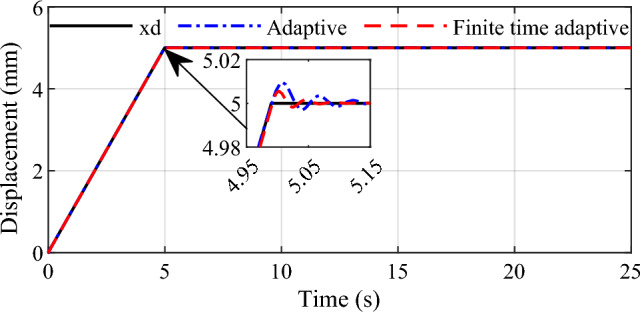
Tracking effect for slope signal input.

**Figure 4:**
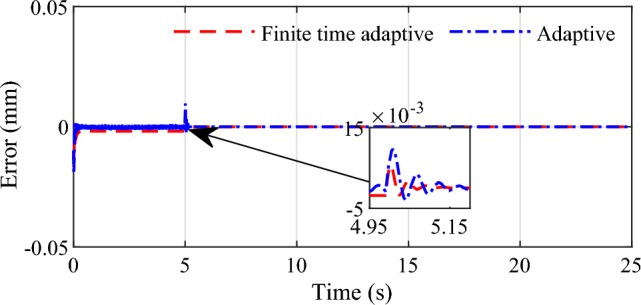
Control error for slope singal input.

In the simulation, the controlled object is the ball screw system combined with the DC motor. The parameters in the simulation system are illustrated as Table 1. According to the established system model and devised position tracking controllers, simulations are carried out as follows. Firstly, slope signal with amplitude 5 is adopted as the input to exhibit the performance of the two control strategies. The simulation results are shown in Figs. 3 and 4.

**Table 1:** Main parameters of the actuator.

Symbol	Description	Value	Unit
$R_{a}$	Armature resistance	0.4933	Ω
$L_{a}$	Back EMF coefficient	0.005	Vs/rad
$K_{e}$	Torque coefficient	0.0189	Nm/A
$K_{t}$	Terminal inductance	0.0187	H
$J_{equ}$	Equivalent moment of inertia	0.000025	kg m^2^
$B_{equ}$	Equivalent damping coefficient	0.0012	Nms/rad
$h$	Screw lead	0.004	m

We can see that the two control methods can achieve good response to slope signal with accurate set point tracking effect. In comparison with the conventional adaptive controller, the finite time adaptive control strategy designed in this paper demonstrates superiority with faster convergence. At the same time, it can be seen that there is smaller overshoot in the tracking response for the finite time adaptive control, which signifies that the proposed method can improve the tracking performance by introducing finite time control idea into conventional adaptive control strategy.

**Figure 5:**
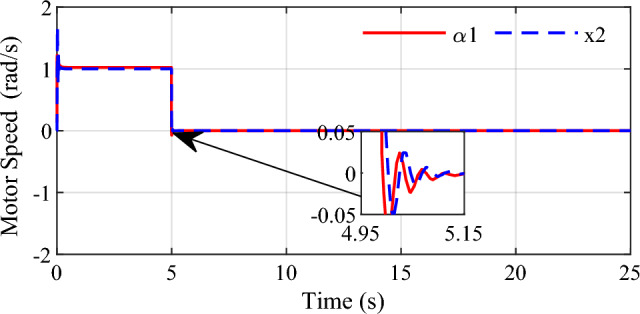
Control law α1 and variable × 2 for slope signal input.

In Fig. 5, it exhibits the simulation result of control rate $\alpha_{1}$ and the speed of the ball screw $x_{2}$. We can see from the Fig. 3 that the displacement of the screw varies continuously with constant slope in the first 5 s, that means the screw velocity is constant which is shown by $x_{2}$ in Fig. 5. When the screw reaches the predetermined shift position at the fifth second, the speed of the screw reduces to zero and there is a small overshoot. From the results we see that the screw speed can better track the changing displacement.

**Figure 6:**
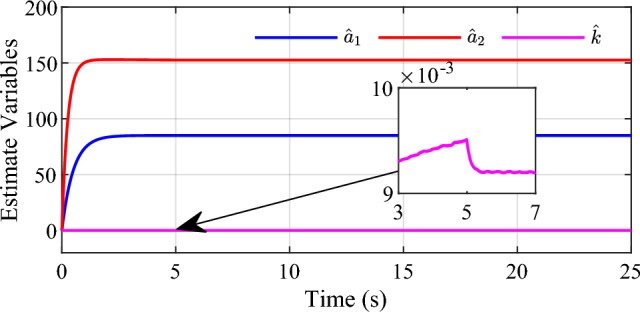
Estimated parameters for slope signal input.

To demonstrate the adaptivity of the estimated parameters in the control system during tracking of slope signal, $\hat{a}_{1}$, $\hat{a}_{2}$ and $\hat{k}$ are selected and illustrated in Fig. 6. We can see that the estimated parameters of the controller are adjusted with the changing expected position, and the estimated parameters become stable subsequently with the steady expected displacement, which indicates that the designed update rate for the parameters is effective.

**Figure 7:**
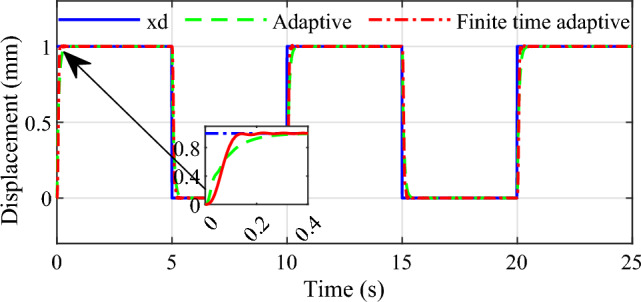
Tracking effect for square wave signal input.

**Figure 8:**
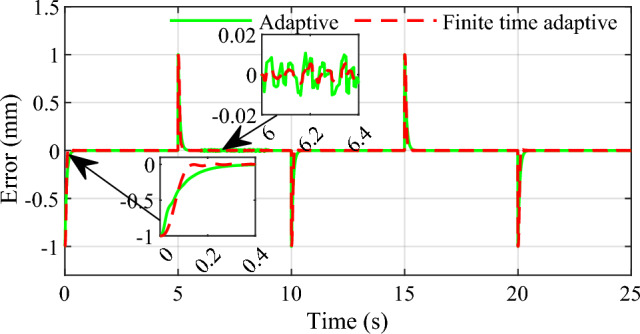
Tracking error for square wave signal input.

Secondly, to demonstrate the set point tracking speed and stability of the controller under relatively extreme conditions, the square wave signal with period of 10 s is designed to simulate the process of continuous shift of automatic transmission, and a system perturbation is added in the 6ths of the simulation to demonstrate the disturbance rejection performance of the system. Similarly, the two control strategies are carried out in the simulations, the results are given in Figs.7 and 8.

From the simulation results we can see that the response speed of the conventional adaptive controller is slow, and the adjust time is about 0.3 s. In comparison, the response speed of the proposed infinite time adaptive controller is faster than that of the conventional adaptive controller, it takes only about 0.15 s, and the strategy tends to stabilization faster than the conventional adaptive control strategy. And when the system is disturbed, the control strategy proposed in this paper has smaller overshoot and more accurate control accuracy than the conventional adaptive control. It is proved that the control scheme designed in this paper is effective and it is able to meet the fast and accurate control requirements for the shifting actuator of AMT automatic transmission.

**Figure 9:**
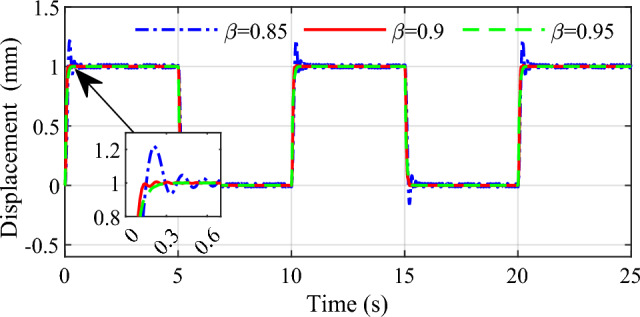
Influence of $\beta$ on the tracking effect.

**Figure 10:**
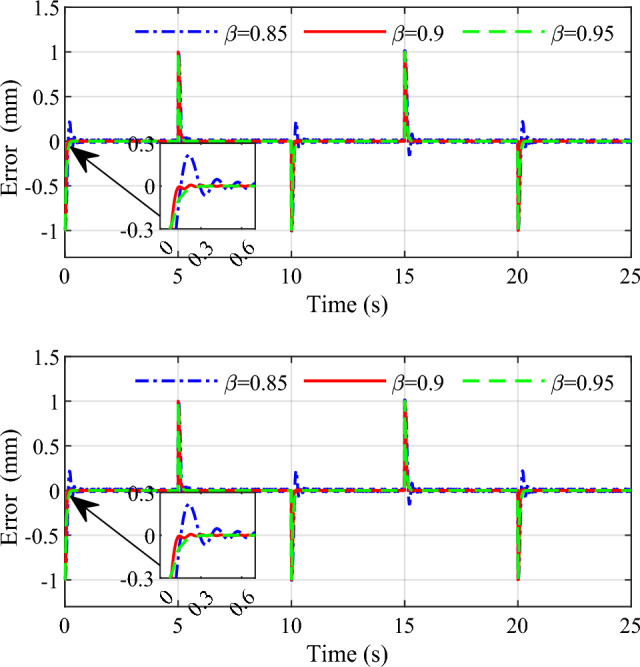
Influence of $\beta$ on the error.

**Figure 11:**
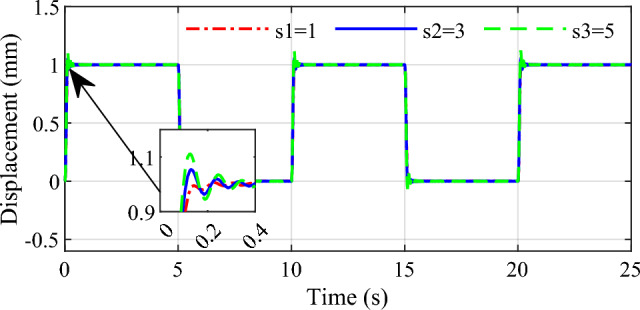
Influence of $s_{1}$ on the tracking effect.

**Figure 12:**
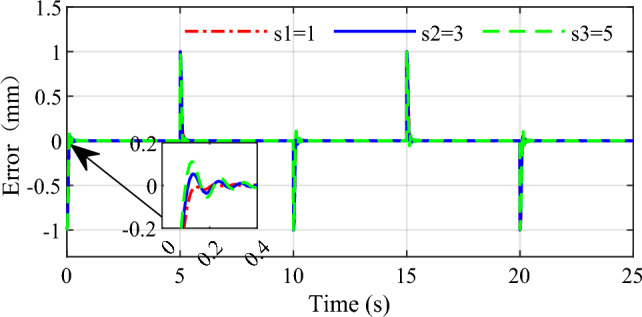
Influence of $s_{1}$ on the error.

In order to demonstrate the influence of the parameters on the control performance, two control parameters $\beta$ and $s_{1}$ are selected in this simulation. Selecting three group values for the two parameters respectively, the response speed and stability to the same input are given in Figs. 9, 10, 11 and 12. From the simulation results we can see that different values of the two parameters both have an impact on the performance of tracking precision and convergence rate.

The values of 0.85, 0.9 and 0.95 are respectively simulated while keeping other parameters consistent, the effect of the parameter $\beta$ is shown in Figs. 9 and 10. As can be seen that the tracking accuracy and convergence rate of the system are better than the other two cases when $\beta$ is set 0.9. Similarly, different values 1, 3 and 5 are set for the parameter $s_{1}$ respectively in Figs. 11 and 12. which shows that good tracking performance is obtained when $s_{1} = 1$, and with the $s_{1}$ increasing, the overshoot of the response is serious accordingly. Adjusting the multiple parameters involved in the controller after a lot of simulations, a set of appropriate parameters are finally determined. The results showed for the square wave signal also prove the validity of the designed parameters.

**Figure 13:**
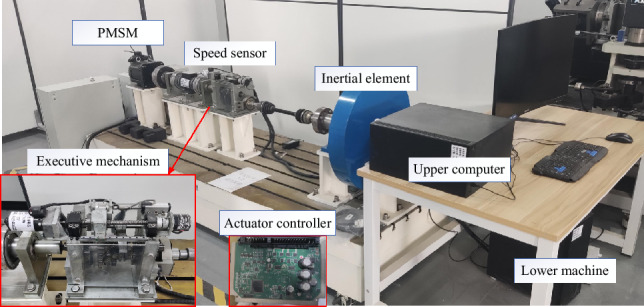
AMT test platform.

From the above simulation results we can see that the presented control scheme exhibits superior position tracking performance. To further verify the feasibility and efficiency of the finite time adaptive control strategy, relevant bench tests and analyses are carried out. The platform for the experimental test is given as Fig. 13.

In the experimental bench, the data processing system is comprised of host computer and slave computer, and CAN bus is adopted to communicate between the two computers. The host computer is responsible for sending out the shift instruction which is transmitted to the actuator controller through the slave computer. To realize gear shift operation, the motor is adjusted by controlling the voltage through the pulse width modulation by the actuator controller. All analog signals are sampled at 1000 Hz through the port of the actuator controller, and digital signals are counted and sampled by the comparator capture unit. These sampled signals are packaged together as CAN signals to transmit to the slave computer through the CAN bus, ultimately to the host computer and processed in the LabVIEW software development environment.

**Figure 14:**
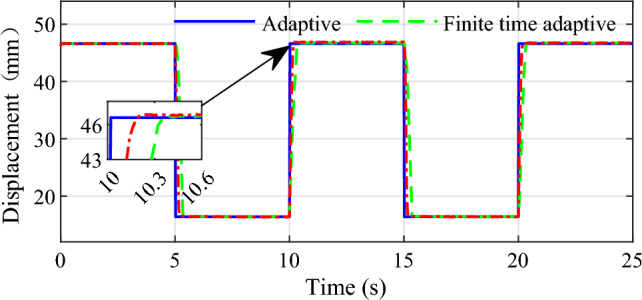
Continuous shift process displacement curve.

**Figure 15:**
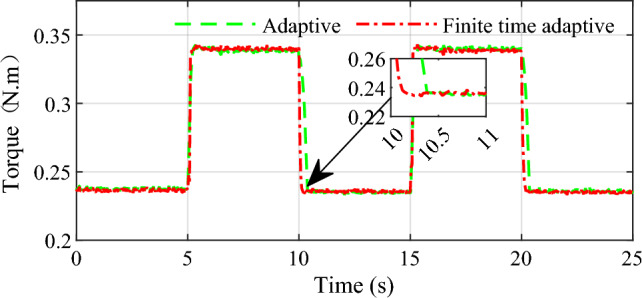
Continuous shift torque curve.

Due to the limitation of experimental equipment, only static AMT gear shift test is carried out on the bench. Therefore, the actuator and computer are the main means considered to execute the static position tracking test. Test will be performed through the continuous shift process between first gear and second gear. As we know, in fact, the processes of shift between different gears are very similar because there is no change for the pattern of sleeve movement, the only changes are the initial rotative speed difference and target gear ratio during synchronization. Referring to the experimental bench parameters, the first gear position at 16.4 mm and the second gear at 46.6 mm for the gear shift actuator. In order to simulate the practical application for the fast and accurate requirements of the shifting process, square wave signal with period of 10 s is used as the desired displacement for the process of shift gear in test. Figures 14 and 15 show the displacement and torque of the shift fork in the process of gear shift respectively.

As can be seen from the experiment results, during the shift between first and second gear, the shift fork can quickly and accurately track the predetermined shift point, as shown in Fig. 14. At the same time, we can see that when shifting from first to second gear, the conventional adaptive control method needs about 0.3 s for the shift fork to reach the specified position, and the finite time adaptive control method based in this paper needs about 0.2 s, which demonstrates superiority in terms of accuracy and rapidity for the proposed method. In the process of continuous shifting, the brushed motor transmits torque to the ball screw and drives the shift fork to realize the transmission gear switching. The torque applied to the shift fork during the continuous shift process is shown in Fig. 15, from which we can see that the control method designed in this paper can enable the shift motor to drive the shift fork to reach the expected position quickly and accurately during the shifting process.

For the bench test, four shifts between the first gear and second gear are accomplished within 25 s. Illustrated by the test results, the designed controller provides precise and fast position tracking performance for the clutch actuator during the continuous shift operation.

## Conclusions

In this paper, a control method combining adaptive control strategy with the finite time idea was proposed to optimize the tracking effect of shifting trajectory, it solved the issue of long gear shift time and poor shift quality in the process of shifting. A shift gear actuator was adopted and gear shift could be realized through screw nut and fork driven by shift motor. A model including the brush motor and the ball screw in the actuator was established. Based on the model, control for the fork displacement was designed by taking merit of the advantage of back stepping and finite time control strategy to accelerate the convergence speed during gear tracking. What’s more, parameter update rate was designed to deal with the model uncertainties and external disturbance. Two kinds of shift signals were used as the desired fork position and the simulation results demonstrated that the shift could be achieved with faster velocity as well as small overshoot, which can meet the requirements of shift gear. The validity of the presented control scheme was further demonstrated on experimental bench. The both results revealed that presented control scheme can realize fast and smooth gear engagement, which gives the vehicle installed AMT a promising prospect with increasing comfortability and drivability.
